# Beyond the coronal plane in robotic total knee arthroplasty—Part 1: Variations in tibial slope and distal femoral flexion do not affect outcomes

**DOI:** 10.1002/ksa.12658

**Published:** 2025-03-25

**Authors:** Luca Andriollo, Christos Koutserimpas, Pietro Gregori, Elvire Servien, Cécile Batailler, Sébastien Lustig

**Affiliations:** ^1^ Department of Orthopaedics Surgery and Sports Medicine FIFA Medical Center of Excellence, Croix Rousse Hospital, Hospices Civils de Lyon, Lyon North University Hospital Lyon France; ^2^ Ortopedia e Traumatologia, Fondazione Poliambulanza Istituto Ospedaliero Brescia Italy; ^3^ Fondazione Policlinico Universitario Campus Bio‐Medico Roma Italy; ^4^ LIBM‐EA 7424, Interuniversity Laboratory of Biology of Mobility, Claude Bernard Lyon 1 University Lyon France; ^5^ Univ Lyon, Claude Bernard Lyon 1 University, IFSTTAR, LBMC UMR_T9406 Lyon France

**Keywords:** combined flexion, functional alignment, functional knee positioning, sagittal alignment, total knee arthroplasty

## Abstract

**Purpose:**

Robotic‐assisted total knee arthroplasty (TKA) and new alignment principles are transforming traditional implant positioning, not only in the coronal plane but also in the sagittal and axial planes. The impact of differences between a patient's native tibial slope (TS) and distal femoral flexion (DFF) compared to the final implant positioning remains unclear. This study aims to evaluate whether variations in TS (ΔTS) and DFF (ΔDFF) play a role in clinical outcomes and implant survival.

**Methods:**

This retrospective study analysed patients who underwent robotic‐assisted TKA following functional alignment (FA) principles between March 2021 and January 2023. A total of 310 patients were included. Preoperative and postoperative data, including radiographic and robotic measurements, were collected. Clinical outcomes (KSS, FJS‐12 and AKPS), range of motion (ROM), complication rates, and implant survival were compared between groups at a minimum of 2 years follow‐up.

**Results:**

At the final follow‐up (mean 2.93 ± 0.62 years), no significant differences were found in clinical scores or ROM between groups with different ΔTS or ΔDFF values. Complication rates and implant survival (99%) were also similar. However, patients with ΔTS > 5° had a slightly increased femoral valgus alignment, while those with ΔDFF ≤ 5° had lower preoperative ROM, with the femoral implant positioned in varus.

**Conclusions:**

Variations between native and implant TS and DFF in robotic‐assisted TKA do not negatively impact functional outcomes or implant survival. A personalised sagittal alignment approach within the concept of FA represents a progression to a functional knee positioning based on three dimensions. Further research is needed to explore the long‐term effects of sagittal alignment on TKA performance.

**Level of Evidence:**

Level III.

**Abbreviations**
AA: anatomical axis
AFO: anterior femoral offset
AKPS: Kujala Anterior Knee Pain Scale
AKPS: Kujala Anterior Knee Pain Scale
AP: anterior–posterior
BMI: body mass index
CR: cruciate‐retaining
CS: cruciate substituting
DAIR: debridement, antibiotics, and implant retention
DFF: distal femoral flexion
FA: functional alignment
FJS‐12: Forgotten Joint Score
FKP: functional knee positioning
HSS: Hospital for Special Surgery Knee Score
KSS: Knee Society Score
LDFA: lateral distal femoral angle
MA: mechanical axis
mHKA: mechanical hip–knee–ankle angle
MPTA: medial proximal tibial angle
PCA: posterior condylar axis
PCL: posterior cruciate ligament
PCO: posterior condylar offset
PS: posterior stabilised
ROM: range of motion
SF‐12: Short Form Health Survey
TEA: transepicondylar axis
TKA: total knee arthroplasty
TS: tibial slope
WOMAC: Western Ontario and McMaster Universities Osteoarthritis Index
ΔDFF: variation in distal femoral flexion
ΔmHKA: difference between pre‐operative and post‐operative mechanical hip–knee–ankle angle
ΔTS: variation in tibial slope

## INTRODUCTION

Robotic surgery and personalised alignment principles are redefining total knee arthroplasty (TKA) positioning by incorporating axial and sagittal variations while respecting native morphology [11, 23, 33, 38].

On the femoral side, the sagittal alignment of the implant plays a fundamental role in both functional performance and the long‐term survival of TKA [10, 43]. Increased extension raises patellofemoral joint forces and anterior knee pain risk, while hyperflexion increases implant failure [17, 21, 29, 36].

The sagittal plane is strongly linked to knee flexion, which is widely recognised as a key factor in determining the success of TKA, as it is essential for many daily activities [19, 24]. Post‐surgical flexion depends on implant sizing, ligament balancing, and osteophyte removal, key factors for optimal outcomes [13, 26].

In conventional knee surgery, the most common recommendation was to position the femoral component at 0°–3° of flexion, based on the distal anatomical axis [18, 44]. However, with robotic‐assisted surgery, which is based on the mechanical axis, distal femoral flexion (DFF) values are more closely correlated with joint functionality, offering a more optimised and personalised approach to alignment.

On the tibial side, the choice of slope is often influenced by the recommendations of implant manufacturers, depending on the type of insert used and the presence of an intrinsic slope in the insert. The tibial slope (TS) is a widely discussed factor that may impact knee flexion angle [14, 22, 42].

Studies on TS highlight its association with knee joint biomechanics, posterior cruciate ligament (PCL) tension and stability, and its role in facilitating femoral rollback. This has implications for clinical outcomes, as an increased TS has been linked to an improved range of flexion while also reducing excessive PCL tension in implants that preserve the ligament, thus lowering the risk of tibial loosening [37]. However, excessive TS can alter knee biomechanics, increasing the risk of anteroposterior instability and potentially reducing implant longevity [22, 30].

In the era of robotic surgery and personalised alignment, it is still unclear whether the difference between native DFF and TS compared to implant positioning has an impact on clinical outcomes and implant survival. The primary objective of this study is to evaluate whether the variation between native TS and implant TS, as well as the variation between native DFF and implant DFF, plays a role in clinical outcomes and implant survival. The hypothesis is that with a personalised alignment, such as functional alignment (FA) or functional knee positioning (FKP), based on three‐dimensional positioning concepts, the sagittal plane, following individualised principles, does not impact clinical outcomes.

## METHODS

This study, designed retrospectively using a prospectively maintained database, included all patients who underwent primary TKA conducted according to FA or FKP principles and performed with the Mako robotic arm‐assisted system (Stryker, Mako Surgical Corp., Fort Lauderdale, FL, USA) between March 2021 and January 2023. All patients received a Triathlon Total Knee System implant (Stryker, Mako Surgical Corp., Fort Lauderdale, FL, USA), using either posterior stabilised (PS) or cruciate substituting (CS) inserts.

**Figure 1:**
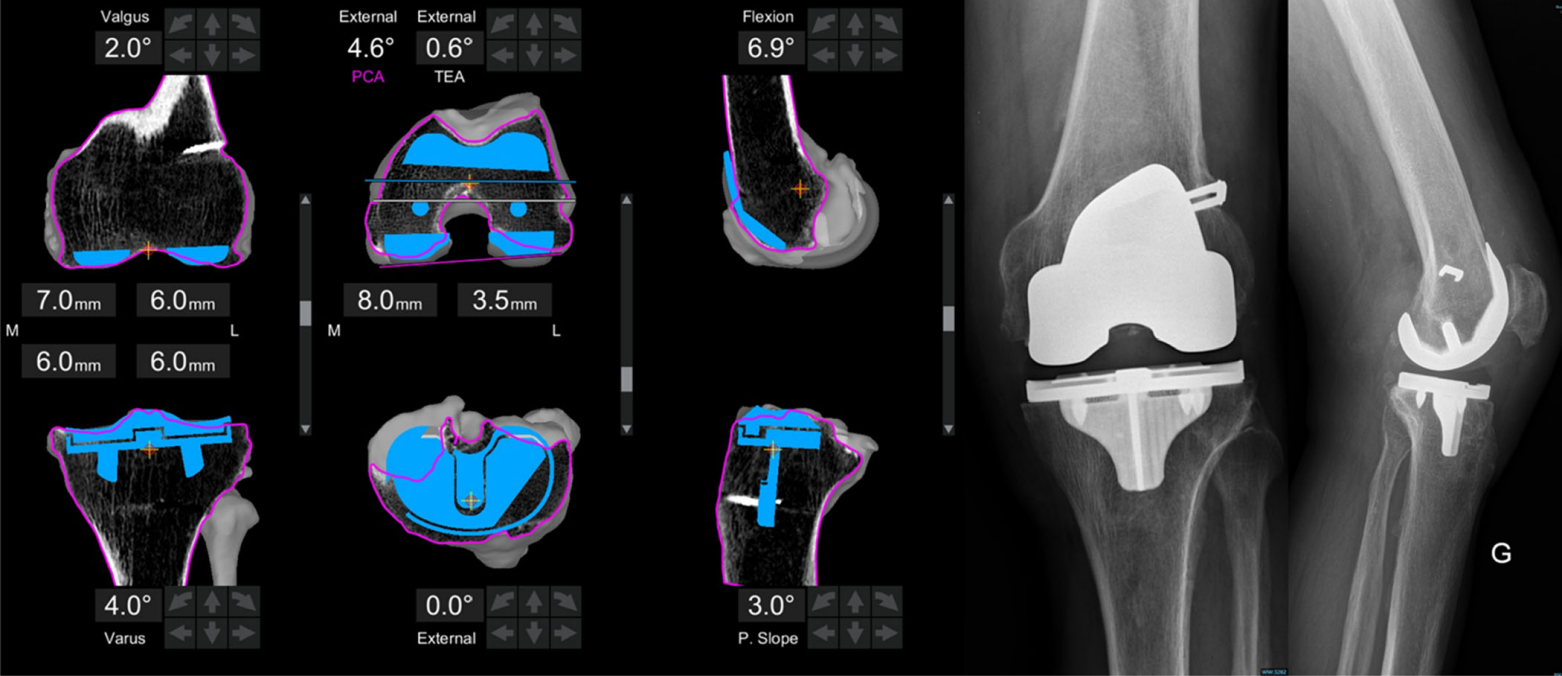
Screenshot from the robotic system of a planning for functional knee positioning following the principles of functional alignment and the related postoperative radiographic evaluation. PCA, posterior condylar axis; TEA, transepicondylar axis.

The procedures were conducted at a single, high‐volume center specialising in both primary and revision arthroplasty. A consistent surgical technique, aligned with FA principles, was applied to all patients [38, 39] (Figure 1).

From the initial pool of 382 patients, those were excluded if preoperative, intraoperative, or follow‐up data required for this study were missing (41 patients), or if a complete robotic data report was unavailable (21 patients).

Demographic data, such as age, gender, and body mass index (BMI), were collected. Preoperative evaluations for all patients included measurements of knee range of motion (ROM) and the Knee Society Score (KSS) for both functional and knee‐specific parameters.

Imaging evaluations were performed using anteroposterior, lateral, Rosenberg, sunrise, and full‐length weight‐bearing X‐rays. From these, the mechanical hip–knee–ankle angle (mHKA), lateral distal femoral angle (LDFA), medial proximal tibial angle (MPTA), and TS (calculated following the mid‐diaphysis technique) were calculated.

Robotic data on component positioning metrics were obtained from robotic system records. For the femoral component, these include flexion/extension based on the mechanical axis (MA), varus/valgus alignment of the distal cut, and rotation relative to the surgical transepicondylar axis (TEA). For the tibial component, varus/valgus alignment of the proximal cut and posterior slope were analysed.

At the final follow‐up, clinical evaluations included KSS‐knee and ‐function, the Forgotten Joint Score (FJS‐12), and the Kujala Anterior Knee Pain Scale (AKPS), as well as data on ROM reported as recurvatum, flexion contracture, and maximum flexion.

Data on complications, both septic and aseptic, as well as reoperation and revision rates, were also collected.

Patients were divided into groups to analyse the impact of implant positioning in relation to the sagittal plane. Specifically, the difference between native data and final implant data was calculated for TS and DFF.

**Figure 2:**
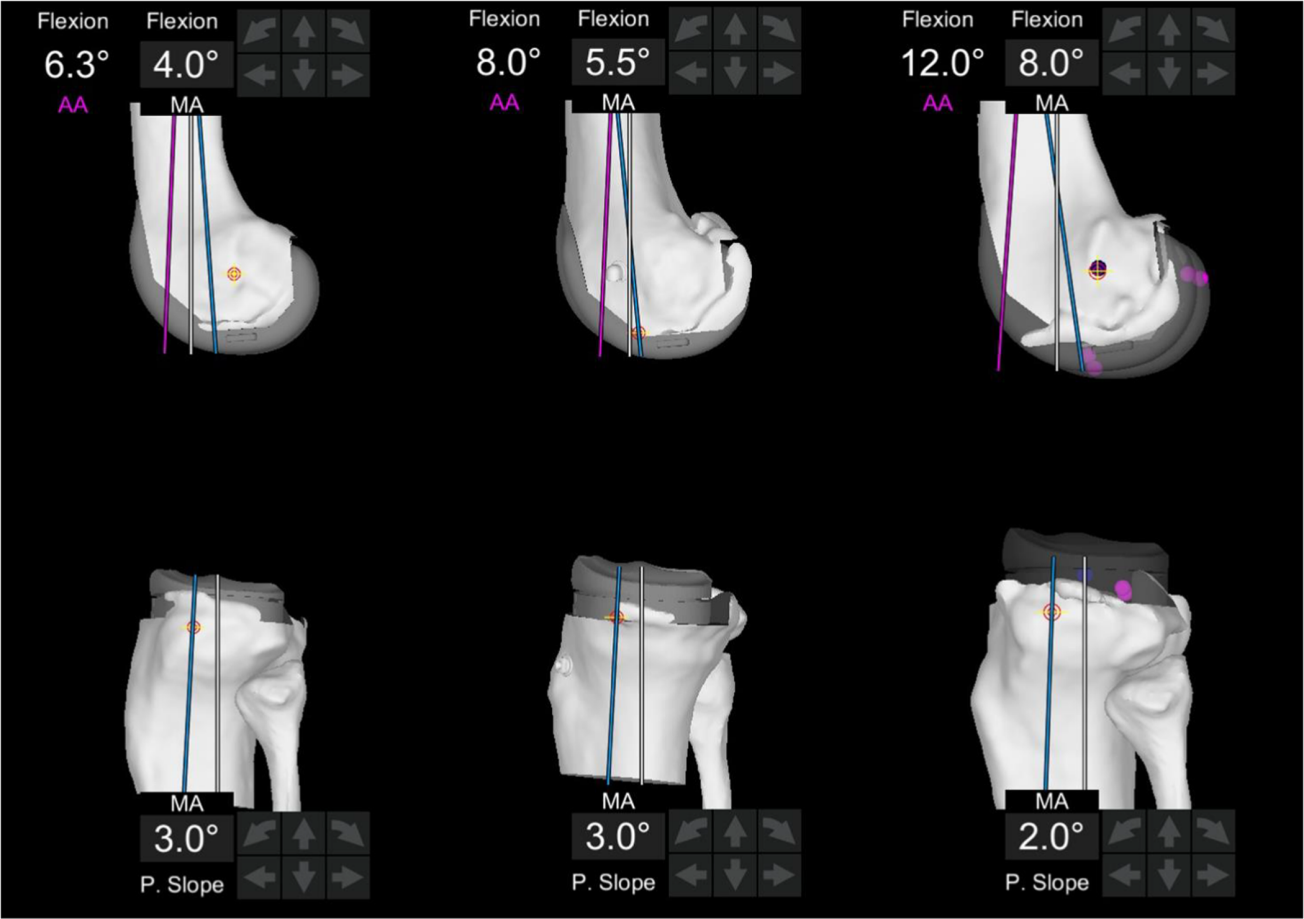
Screenshots from the robotic system displaying the anatomical axis (AA, pink line), mechanical axis (MA, white line), and implant axis (blue line).

The native TS slope, calculated from X‐rays, was compared to the implant slope, obtained from robotic data, resulting in an ΔTS value (ΔTS = native TS − implant TS). Regarding the femur, it is known that the native DFF is the angle between the anatomical axis and the MA. The final femoral flexion of the implant, provided by the robot, is the angle between the MA and the implant axis. Consequently, by definition, the final femoral flexion of the implant represents the variation angle of the DFF, with reference to the anatomical axis (Figure 2). The femoral flexion value provided by the robot will hereafter be identified as ΔDFF, indicating the difference in DFF between the native and final states.

Positive (+) values of ΔTS indicate that the native TS was greater than the implant TS, and positive (+) values of ΔDFF represent an increase in femoral flexion.

Based on ΔTS, the group with values between 0° and 5° degrees (Group A) were compared to the group with values > 5° (Group B). Regarding ΔDFF, the group with values ≤ 5° (Group C) was compared to the group with values > 5° (Group D).

The groups, within the two respective analysed parameters, were compared in terms of clinical outcomes, radiographic measurements, robotic data and complication rates.

### Principles of sagittal plane management

The femoral component is placed to closely match the concentricity of Blumensaat's line in cases without trochlear dysplasia while avoiding femoral notching. This method ensures proper component sizing and reduces the risk of patellofemoral overstuffing or understuffing. Femoral flexion ranged from 0° to 10°.

The tibial implant is positioned to replicate the patient's native posterior TS, with a maximum limit of 3° when using a PS implant. Adjustments can be made to balance the flexion gap if needed, while ensuring that the combined femoral‐tibial flexion does not exceed 10°.

The PS insert was preferred in cases of posterior cruciate ligament (PCL) deficiency or significant flexion contracture. With the CS insert, PCL release is performed only in cases of severe flexion limitation with the trial component, but it remains rare.

### Ethical approval

This study followed the ethical guidelines established in the 1964 Declaration of Helsinki and complied with HIPAA standards. The collection and analysis of data were carried out in alignment with the MR004 Reference Methodology set by the French Commission Nationale de l'Informatique et des Libertés (Ref. 2229975V0). Informed consent was obtained from all participants.

### Statistical analysis

A power analysis was conducted using G*Power (*α* = 0.05, *β* = 0.20, medium effect size), identifying a minimum of 72 patients per group was determined to be necessary for comparing variables. All the groups in this study meet the minimum sample. Continuous variables were expressed as mean and standard deviation (SD), while categorical variables were presented as frequency distributions and percentages. The Shapiro–Wilk test was employed to evaluate the normality of the data. For comparisons between two groups, either the *t*‐test or the Mann–Whitney *U* test was used, depending on whether the data followed a normal distribution. Categorical variables were analysed using the chi‐square test.

A 95% confidence interval was applied, and statistical significance was defined as a *p*‐value < 0.05. All statistical analyses were performed using Python version 3.11 (Python Software Foundation, Wilmington, DE, USA) and the stats models library (v0.13).

## RESULTS

At the final follow‐up, 310 patients were evaluated with a mean follow‐up period of 2.93 ± 0.62 years.

The cohort included 181 females (58.4%) and 139 males (41.6%), with an average age of 69 ± 8.3 years. A total of 167 right knees (53.9%) and 143 left knees (46.1%) were treated. The mean BMI was 28.6 ± 4.9 kg/m². Among the patients, a CS liner was used in 107 cases (34.5%), while a PS liner was used in 203 cases (65.5%).

At the preoperative clinical evaluation, the mean KSS‐knee score was 64.5 ± 13.1, and the KSS‐function score was 67.9 ± 15.3. Regarding the ROM data, the mean recurvatum was 0.6° ± 2.2°, the mean flexion contracture was 2.2° ± 4°, and the mean maximum flexion was 119.3° ± 11.8°.

In the preoperative radiographic assessment, the mean mHKA was 174.1° ± 5.5°, the LDFA was 89.3° ± 5.8°, the MPTA was 86.3° ± 3.1°, and the TS was 7.4° ± 3.1°. The Mako data on tibial component positioning showed an average varus alignment of 3.3° ± 1.7° and an average slope of 0.8° ± 0.8°. For the femoral component, the mean valgus was 0.5° ± 1.8°, the average implant flexion (ΔDFF) was 6.9° ± 2.6°, and the mean external rotation was 0.2° ± 1.85°. The average ΔTS was 6.6° ± 3.1°.

At the final follow‐up, 21 major complications were reported, corresponding to 6.8% and representing the reintervention rate. Specifically, 13 patients (4.2%) underwent manipulation under anaesthesia (MUA) or arthroscopic arthrolysis within the first 6 months after first surgery due to stiffness, three patients (1%) underwent surgery for revision of the surgical scar, two patients (0.6%) were reoperated on for acute infection using the DAIR technique (debridement, antibiotics and implant retention), one patient (0.3%) underwent reoperation for revision of the femoral component, one patient (0.3%) underwent revision of the patellar component and one patient (0.3%) underwent a complete revision due to previously unknown nickel allergy.

The final implant survival rate at the final follow‐up was 99%, with 1% of patients undergoing partial or total implant revision.

### Variation in TS

Group A consisted of 110 patients (35.5%) and Group B of 200 patients (64.5%). In the comparison between these two groups, no statistically significant differences were found in terms of mean follow‐up (*p* = 0.32), gender (*p* = 0.43), age (*p* = 0.11), side (*p* = 0.56) and BMI (*p* = 0.31). The type of liner used showed a significant difference (*p* = 0.03), with the CS liner used in 47 patients (42.7%) in Group A and in 60 patients (30%) in Group B.

No preoperative differences were observed in terms of clinical scores, specifically in KSS‐knee (*p* = 0.39), KSS‐function (*p* = 0.29) and ROM (recurvatum: *p* = 0.64; flexion contracture: p = 0.56; maximum flexion: *p* = 0.74). Similarly, the preoperative radiographic analysis revealed no statistically significant differences in mHKA (*p* = 0.8), LDFA (*p* = 0.67) and MPTA (*p* = 0.36). Regarding Mako data, there were no differences in tibial varus (*p* = 0.62), femoral external rotation (*p* = 0.41) and ΔDFF (*p* = 0.11). However, a statistically significant difference was observed in femoral valgus (*p* = 0.04), with Group A having a mean of 0.3° ± 1.8°, lower than 0.7° ± 1.8° in Group B.

**Figure 3:**
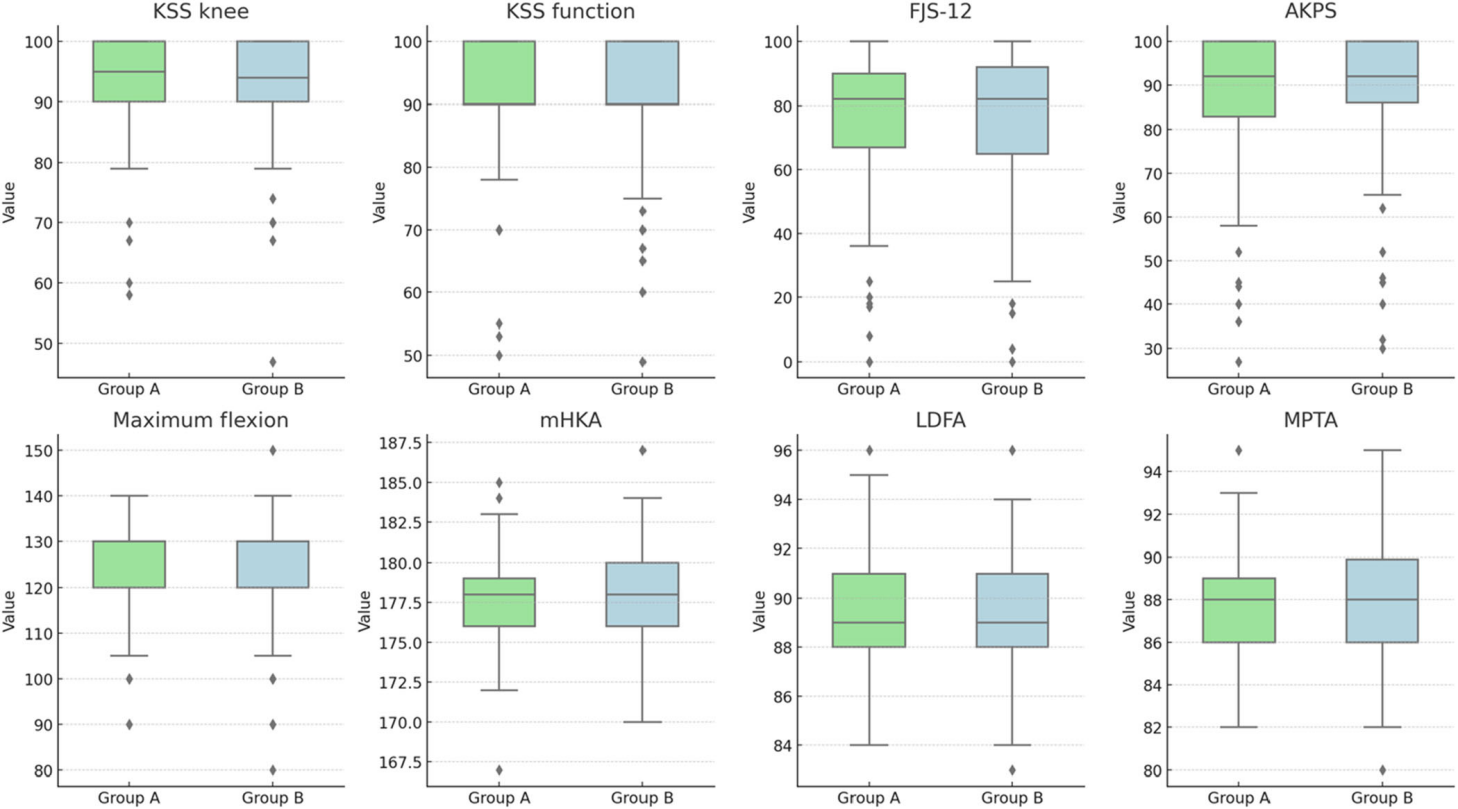
Graphical representation with box plots of the parameters analysed in the comparison between Group A and Group B, categorised based on ΔTS, which represents the variation between native and implant tibial slope (TS). Group A includes patients with values between 0° and 5°, while Group B includes patients with ΔTS > 5°. AKPS, Kujala Anterior Knee Pain Scale; FJS‐12, Forgotten Joint Score; KSS, Knee Society Score; LDFA, lateral distal femoral angle; mHKA, mechanical hip–knee–ankle angle; MPTA, medial proximal tibial angle.

At the final follow‐up, no differences were observed in clinical outcomes or radiographic measurements. The details are provided in Table 1. Figure 3 shows the box plots of the analysed parameters.

**Table 1:** Comparison of clinical outcomes and radiographic measurements at final follow‐up between Group A and Group B, categorised based on ΔTS, which represents the variation between native and implant tibial slope (TS).

	Group A (*N* = 110)	Group B (*N* = 200)	*p* value
KSS knee	92.9 (SD 8.4)	92.8 (SD 8)	0.86
KSS function	91.4 (SD 10.2)	92.2 (SD 8.8)	0.49
Recurvatum (degrees)	1.1 (SD 2.4)	1.1 (SD 2.5)	0.85
Flexion contracture (degrees)	0.2 (SD 1.1)	0.2 (SD 1.3)	0.92
Maximum flexion (degrees)	124.4 (SD 10.3)	124.4 (SD 10.4)	0.98
FJS‐12	74.8 (SD 24.3)	77.5 (SD 20.5)	0.33
AKPS	88.1 (SD 15.2)	89.4 (SD 12.9)	0.44
mHKA (degrees)	177.9 (SD 2.8)	177.9 (SD 3)	0.83
LDFA (degrees)	89.4 (SD 2.2)	89.3 (SD 2.5)	0.78
MPTA (degrees)	88 (SD 2.3)	87.9 (SD 2.3)	0.82

*Note*: Group A includes patients with values between 0° and 5°, while Group B includes patients with ΔTS > 5°.

Abbreviations: AKPS, Kujala Anterior Knee Pain Scale; FJS‐12, Forgotten Joint Score; KSS, Knee Society Score; LDFA, lateral distal femoral angle; mHKA, mechanical hip–knee–ankle angle; MPTA, medial proximal tibial angle; SD, standard deviation.

There was no statistically significant difference in the overall complication rate (6.4% vs. 7.0%; *p* = 0.83), nor specifically in the causes of mechanical failure (4.5% vs. 5.0%; *p* = 0.86), the reoperation rate (5.5% vs. 6.0%; *p* = 0.85), or the implant revision rate (0.9% vs. 1.0%; *p* = 0.94).

### Variation in DFF

Group C consisted of 73 patients (23.5%) and Group D of 237 patients (76.5%). In the comparison between these two groups, specifically between patients with ΔDFF ≤ 5° and those with ΔDFF > 5°, no statistically significant differences were found in terms of mean follow‐up (*p* = 0.41), gender (*p* = 0.56), age (*p* = 0.81), side (*p* = 0.73), BMI (*p* = 0.15) and type of insert (*p* = 0.19).

No preoperative differences were observed in KSS‐knee (*p* = 0.29), KSS‐function (*p* = 0.18) and recurvatum (*p* = 0.64). However, a statistically significant difference was found in flexion contracture (*p* = 0.002), with Group C showing higher values (3.2° ± 4.4°) compared to Group D (1.9° ± 3.8°). Similarly, a statistically significant difference was observed in maximum flexion (*p* = 0.01), with Group C showing lower values (116.1° ± 11.9°) compared to Group D (120.3° ± 11.6°).

In the preoperative radiographic evaluation, the mHKA in Group C was 172.9° ± 4.5°, while in Group D it was 174.5° ± 5.8°, showing a significant difference (*p* = 0.02). No significant differences were found for LDFA (*p* = 0.08), MPTA (*p* = 0.63) and native TS (*p* = 0.14).

Regarding Mako data, there were no differences in tibial varus (*p* = 0.53) and femoral external rotation (*p* = 0.19). However, a statistically significant difference was observed in femoral varus/valgus (*p* = 0.001), with Group C having a mean of −0.19° ± 2.2° (0.19° varus), while Group D had a valgus of 0.77° ± 1.66°. Additionally, a statistically significant difference was observed in TS (*p* = 0.002), with Group C having a mean of 1.01° ± 0.84°, higher than Group D, which had 0.69° ± 0.79°.

**Figure 4:**
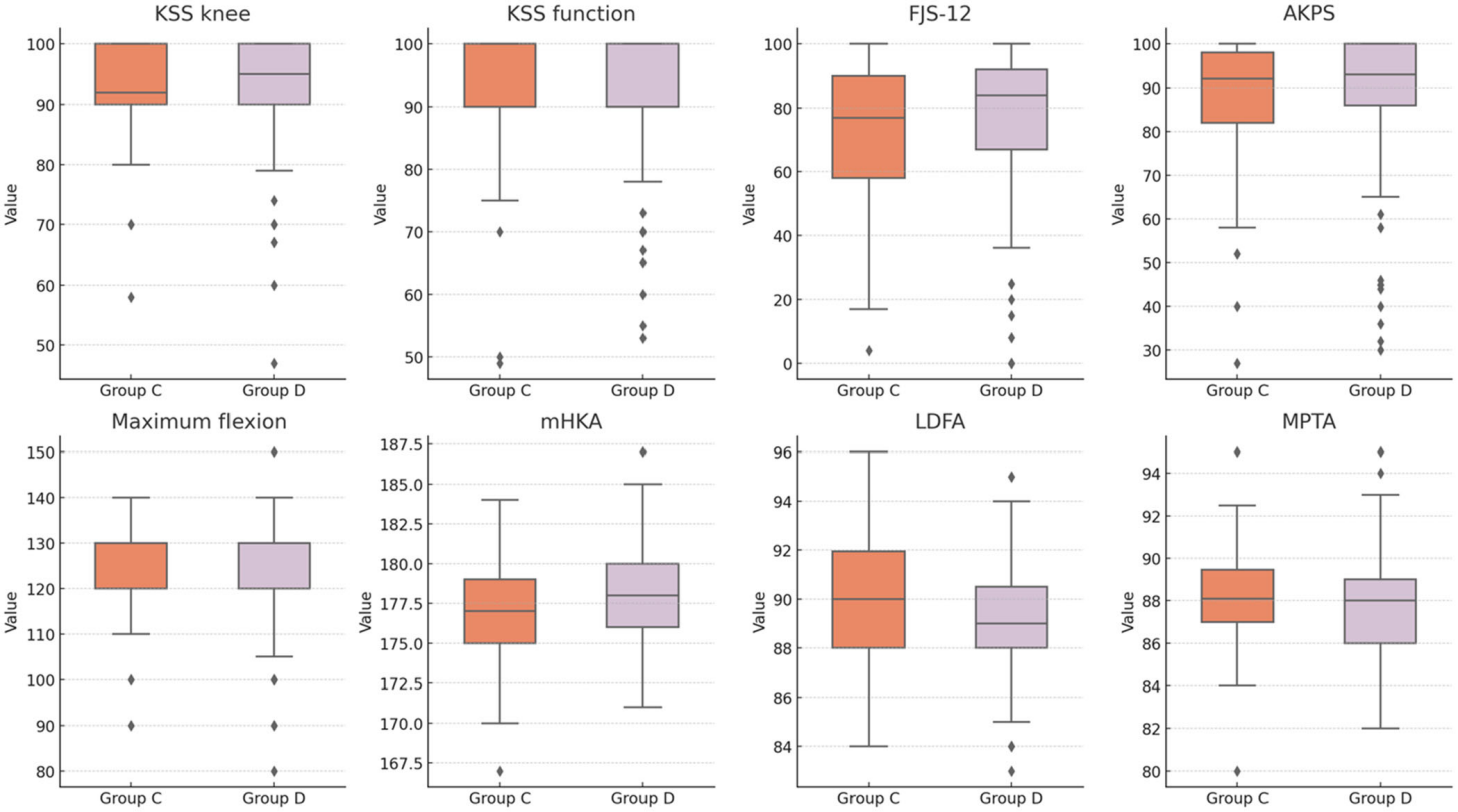
Graphical representation with box plots of the parameters analysed in the comparison between Group C and Group D, categorised based on ΔDFF, which represents the variation between native and implant distal femoral flexion. Group C includes patients with ΔDFF ≤ 5°, while Group D includes patients with ΔDFF > 5°. AKPS, Kujala Anterior Knee Pain Scale; FJS‐12, Forgotten Joint Score; KSS, Knee Society Score; LDFA, lateral distal femoral angle; mHKA, mechanical hip–knee–ankle angle; ΔmHKA: difference between pre‐operative and post‐operative mechanical hip–knee–ankle angle; MPTA, medial proximal tibial angle.

At the final follow‐up, no differences were observed in clinical outcomes or radiographic measurements, except for post‐operative mHKA (*p* = 0.02). Considering the pre‐operative mHKA difference, this parameter was further investigated by evaluating the difference between pre‐operative and post‐operative values (ΔmHKA), showing no statistical significance (*p* = 0.49). The details are provided in Table 2. Figure 4 shows the box plots of the analysed parameters.

**Table 2:** Comparison of clinical outcomes and radiographic measurements at final follow‐up between Group C and Group D, categorised based on ΔDFF, which represents the variation between native and implant distal femoral flexion.

	Group C (*N* = 73)	Group D (*N* = 237)	*p* value
KSS knee	92.4 (SD 8.2)	92.9 (SD 8.2)	0.58
KSS function	91 (SD 10)	92.2 (SD 9)	0.29
Recurvatum (degrees)	1.3 (SD 2.9)	1 (SD 2.3)	0.69
Flexion contracture (degrees)	0.2 (SD 1.1)	0.2 (SD 1.2)	0.67
Maximum flexion (degrees)	123.2 (SD 10.3)	124.8 (SD 10.3)	0.16
FJS‐12	72.1 (SD 24.2)	77.9 (SD 21.1)	0.06
AKPS	87.4 (SD 14.9)	89.4 (SD 13.4)	0.28
mHKA (degrees)	177 (SD 3.1)	178.1 (SD 2.9)	0.02
ΔmHKA (degrees)	−3.9 (SD 3.9)	−3.5 (SD 4.6)	0.49
LDFA (degrees)	89.9 (SD 2.8)	89.1 (SD 2.2)	0.09
MPTA (degrees)	88.1 (SD 2.3)	87.9 (SD 2.3)	0.47

*Note*: Group C includes patients with ΔDFF ≤ 5°, while Group D includes patients with ΔDFF > 5°.

Abbreviations: AKPS, Kujala Anterior Knee Pain Scale; FJS‐12, Forgotten Joint Score; KSS, Knee Society Score; LDFA, lateral distal femoral angle; mHKA, mechanical hip–knee–ankle angle; ΔmHKA: difference between pre‐operative and post‐operative mechanical hip–knee–ankle angle; MPTA, medial proximal tibial angle.

There was no statistically significant difference in the overall complication rate (5.5% vs. 7.2%; *p* = 0.62), nor specifically in the causes of mechanical failure (5.5% vs. 4.6%; *p* = 0.78), the reoperation rate (4.1% vs. 6.3%; *p* = 0.49), or the implant revision rate (1.4% vs. 0.84%; *p* = 0.68).

## DISCUSSION

The main findings of this study indicate that there is no statistically significant clinical difference between patients with different ΔTS, which represents the difference between the native TS and the implant slope. The only observed difference is that, following the principles of FA and to achieve the desired balance in extension and 90° flexion on the coronal plane, the femur was positioned with greater valgus in the group of patients with ΔTS > 5°.

At the femoral level, ΔDFF was analysed, representing the difference between native flexion and implant flexion. Patients who, according to alignment and balancing principles, required a ΔDFF ≤ 5° had a preoperative condition of reduced ROM, both in terms of flexion contracture and maximum flexion. Additionally, in this group of patients, the robotic positioning of the femoral component was in varus, with a significant difference compared to the group with ΔDFF > 5°, where the femoral component was positioned in valgus, and the tibial component positioning showed a significantly increased TS. At both the femoral and tibial sides, no significant differences were found regarding complications.

In recent years, new principles of coronal alignment have been proposed, differing from the traditional mechanical alignment. However, despite enabling technologies, the use of robotic surgery, and the resulting increase in surgical precision, the optimal sagittal alignment has not yet been defined [2, 10, 15]. The use of robotic technology has improved the accuracy of prosthetic implant positioning [34, 41]. In contrast, conventional techniques have shown suboptimal results in achieving the desired femoral flexion positioning, with success observed in only 41.3% of cases [43]. In a recent CT scan study, the difference between the axes used in conventional technique and those calculated in the robot‐assisted technique was evaluated [1].

In conventional techniques, sagittal implant alignment relies on limited anatomical landmarks that are palpable during surgery and determined intraoperatively using intramedullary or extramedullary rods. In contrast, robotic systems base alignment on the MA of the entire femur and tibia. This approach results in 1.4° less femoral component flexion compared to techniques that reference the distal intramedullary canal [6].

On the femoral side, the robotic MA was found to be 2.5° more extended compared to the distal femoral intramedullary axis. On the tibial side, the robotic MA was found to be 2.4° more flexed compared to the manual intramedullary axis.

Moreover, the robotic technique achieved better accuracy than the conventional group in terms of sagittal plane positioning (*p* < 0.05) [27].

Most TKA implant manufacturers recommend specific tibial resection slopes for their systems. However, these recommendations may not always align with a patient's native TS, which varies significantly among individuals. Many prosthetic designs aim for a tibial sagittal alignment between 0° and 7°, achieved either through bone cuts or by incorporating an intrinsic slope in the polyethylene liner [3].

Cadaveric studies have shown that for every 1° increase in TS, there is an average increase of 1.7° in knee flexion [4]. This occurs due to the delayed impingement of the tibial insert on the femoral bone.

However, clinical studies investigating the correlation between TS and knee function have yielded mixed results. Antony et al. analysed 105 cruciate retaining (CR) TKA cases and found no significant correlation between TS and ROM, maximum extension, or maximum flexion [3]. Similarly, Miralles–Muñoz assessed preoperative and postoperative ΔTS, reporting no impact on functional outcomes at 5 years, as measured by the KSS, the Western Ontario and McMaster Universities Osteoarthritis Index (WOMAC), and ROM [25]. Comparable results, meaning no differences in terms of KSS, WOMAC, and ROM, have been reported in further studies on both CR and PS TKA [8, 22]. Sinno et al. evaluated 168 PS TKA patients using Short Form Health Survey (SF‐12) mental and physical scores and functional KSS. They found that in cases where TS was greater than 5°, functional outcomes were similar between PS and CR implants, while they observed inferior outcomes in patients with PS TKA and TS < 5° [40].

Despite numerous studies reporting no role of TS in clinical outcomes, some studies have identified significant differences. Seo et al. [37], in a study of 801 CR TKA cases, observed no differences in KSS knee and functional scores or ROM across different TS groups. However, they reported better Feller patella scores and Kujala scores (AKPS) in patients with ΔTS between −1° and 3° [37]. Pan et al. [31] examined CR TKA patients with ΔTS greater than 3° and those with changes ≤ 3°, reporting better KSS, WOMAC, and pain scores in patients with greater slope variation. Lee et al. [22] analysed 164 knees and found no alterations in ROM, KSS, or FJS based on TS changes. However, multivariate analysis revealed a weak positive correlation with “difficulty in rising from sitting” (WOMAC) and “awareness when climbing stairs” (FJS) [22].

Regarding complications, Kim et al. [20] reported increased failure rates in patients with a tibial component malalignment (<0° or >7°). Nedopil also suggested that excessive TS may increase the risk of tibial component loosening, particularly due to anteroposterior tilt failure [28]. However, this risk may depend on implant design. Fujito et al. [12], in a study of 71 CR TKA cases with high geometric conformity to the medial articular surface, found no significant differences in complications, even in patients with TS up to 10°. Similarly, Richardson et al. [32], in a radiostereometric analysis of 200 TKA cases, found no association between postoperative TS and overall migration, anteroposterior tilt migration, or inducible displacement.

The optimal sagittal alignment of the femoral component in TKA remains under investigation, particularly when comparing the efficacy of a default position versus an individualised approach [5].

Wang et al. [43] conducted a study on 120 knees that underwent robotic‐assisted TKA, dividing them into two groups: one with individualised femoral flexion and the other with a default flexion of 3°–5°. Their findings indicated that the individualised alignment group had significantly lower incidences of femoral prosthesis extension and a higher rate of optimal prosthesis flexion between 0° and 3°.

The importance of sagittal femoral alignment in improving postoperative function is well documented. Nishitani et al. [29] reported that patients with mildly flexed femoral prostheses achieved higher functional scores 1 year after TKA compared to those with extended or hyperflexed prostheses. Similarly, Hassan et al. [9] found that, after a 2‐year follow‐up, femoral prosthesis flexion of 0°–3° was associated with a knee that felt “always normal”.

Murphy et al. [26] conducted a double‐blind, randomised controlled trial on CR TKA patients, comparing femoral components positioned at 0° and 4°. They assessed knee flexion, knee extension, WOMAC, SF‐12, the timed stand test, the stair climb test, and overall patient satisfaction at one year postoperatively. The only significant differences were improved knee flexion and SF‐12 scores in the group with the femoral component positioned at 4°.

Antony et al. [3] reported a weak positive correlation between femoral component flexion and both maximum knee flexion and ROM. However, increased failure rates have been observed in knees where the femoral component was flexed beyond 3°. While increased flexion may improve knee flexion outcomes, the heightened risk of early aseptic failure outweighs the potential clinical benefits.

Chen et al. [7] analysed the impact of femoral component positioning at 0°, 3°, and 6° of flexion in the sagittal plane. They observed that a 3° flexion implantation could reduce excessive overstuffing, although 3.10 mm of overstuffing remained at the medial side of zone 1. Conversely, 6° flexion could eliminate 3 mm of overstuffing across all zones but increased the risk of understuffing. Thus, slight femoral flexion may be a useful technique to prevent excessive component overstuffing, particularly in the trochlea and anterior region of the distal condyle.

The positioning and alignment of the femoral component in the sagittal plane have been associated with long‐term anterior knee pain, with femoral component extension being a significant risk factor [35].

This study has some limitations. The design is retrospective, and the follow‐up period is relatively short. The use of a single implant type and a single robotic system may limit the applicability of the results to other designs or platforms. The inter‐ and intra‐observer reliability of radiographic measurements was not assessed. A more detailed analysis of variations of knee phenotypes should be conducted to obtain more personalised insights [16]. The follow‐up time, although relatively short, is sufficient for assessing short‐term outcomes but may limit considerations regarding complications. The radiographic evaluation did not include the assessment of radiographic signs of early loosening.

Despite these limitations, the study provides insights into sagittal alignment in robotic‐assisted TKA. Future research with longer follow‐up and advanced biomechanical analysis will help refine these findings and further clarify the long‐term impact of sagittal alignment variations on clinical outcomes and implant longevity. Furthermore, future research should evaluate the relationship between sagittal plane variations and implant cuts, which have shown significant differences.

## CONCLUSION

This study highlights that personalised sagittal alignment, following the principles of FA in robotic‐assisted TKA, does not negatively impact outcomes or mid‐term implant survival. Differences between native and implanted TS and DFF are the result of specific positioning adjustments and did not lead to significant differences in functional scores, ROM or complication rates. Using a personalised approach to sagittal alignment follows the idea of adjusting implant positioning to match each patient's natural anatomy.

The role of the sagittal plane will be further explored in the second part of this study, titled “Beyond the coronal plane in robotic total knee arthroplasty – Part 2: Combined flexion does not affect outcomes”.

## AUTHOR CONTRIBUTIONS

Luca Andriollo and Sebastien Lustig had the idea for the article. Luca Andriollo was responsible for writing of the manuscript and qualified as corresponding author. Pietro Gregori was responsible for data acquisition and analysis and realisation of Figures and Tables. Christos Koutserimpas was responsible of statistical analysis. Cécile Batailler and Elvire Servien were responsible for conceptualisation and supervised data acquisition and analysis. Cécile Batailler, Elvire Servien and Sebastien Lustig were responsible for reviewing and critically revise the manuscript. All authors have given final approval of the version to be published.

## CONFLICT OF INTEREST STATEMENT

Luca Andriollo and Christos Koutserimpas have nothing to declare. Cécile Batailler: Consultant for Smith and Nephew and Stryker. Elvire Servien: Consultant for Smith and Nephew. Sébastien Lustig: Consultant for Heraeus, Stryker, Depuy Synthes, Smith and Nephew. Institutional research support to Lepine and Amplitude.

## ETHICS STATEMENT

This study adhered to the ethical principles outlined in the 1964 Declaration of Helsinki and complied with HIPAA regulations. Data collection and analysis were conducted in accordance with the MR004 Reference Methodology of the French Commission Nationale de l'Informatique et des Libertés (Ref. 2229975V0). All patients provided legitimate informed consent.

## Data Availability

The data that support the findings of this study are available from the corresponding author, [L.A.], upon reasonable request.
